# Stress Analysis and Strength Prediction of Carbon Fiber Composite Laminates with Multiple Holes Using Cohesive Zone Models

**DOI:** 10.3390/polym17010124

**Published:** 2025-01-06

**Authors:** Hamzah Alharthi, Mohammed Y. Abdellah

**Affiliations:** 1 Mechanical Engineering Department, College of Engineering and Architecture, Umm Al-Qura University, Makkah 21955, Saudi Arabia; haharthi@uqu.edu.sa; 2 Mechanical Engineering Department, Faculty of Engineering, South Valley University, Qena 83523, Egypt; 3 Mechanical Engineering Department, College of Engineering, Alasala University, Dammam 31483, Saudi Arabia

**Keywords:** cohesive zone, damage, carbon fiber, stress intensity factor

## Abstract

Composite materials play a crucial role in various industries, including aerospace, automotive, and shipbuilding. These materials differ from traditional metals due to their high specific strength and low weight, which reduce energy consumption in these industries. The damage behavior of such materials, especially when subjected to stress discontinuities such as central holes, differs significantly from materials without holes. This study examines this difference and predicts the damage behavior of carbon fiber composites with multiple holes using a progressive damage model through finite element analysis (FEM). Two holes were positioned along the central axis of symmetry in the longitudinal and transverse directions relative to the load. The presence of additional holes acts as a stress-relief factor, reducing stress by up to 17% when the holes are arranged in the longitudinal direction. A cohesive zone model with two parameters, including constant and linear shapes, was applied to develop a simple analytical model for calculating the nominal strength of multi-hole composite laminates, based on the unnotched plate properties of the material. The results closely match experimental findings. The data also provide design tables that can assist with material selection.

## 1. Introduction

Composite laminate structures play a key role in various industrial applications, such as the automotive, aerospace, and marine industries. This is because of their advantageous properties, including low specific weight, excellent resistance to corrosion and erosion, and high fracture toughness under load [1].

The nominal strength of composite structures with open holes is affected by the size effect phenomenon [1], which refers to a reduction in strength as the structure’s geometry is scaled [2]. The nominal strength of composite laminates is influenced by factors such as hole size [3], hole shape [4], and orientation [5,6]. Mohammed [7] simulated the nominal strength of composite laminates with central holes using cohesive laws with two parameters. The study found that the shape of the holes plays a significant role in strength performance, with the constant cohesive law having a greater effect on the fracture toughness (G_IC_) compared to the linear and exponential cohesive laws.

The performance of composite laminates was enhanced by introducing additional holes in the longitudinal direction [8]. This improvement is attributed to a reduction in stress concentration around the central holes when two holes are present. However, the failure modes in laminates with two holes include delamination and matrix failure, which differ from the behavior observed with a single hole.

A 2D finite element analysis (FEA) was conducted on a woven composite plate with multiple holes. The study revealed that, in non-staggered configurations, the maximum stress at the outer holes causes failure of the net cross-sectional area, whereas staggered configurations depend on the smallest net cross-sectional area. The numerical modeling showed good agreement with the experimental results, accurately predicting crack locations and stress behavior [9]. The arrangement of the holes significantly affects the strength of composite plates. Plates with double-row configurations have smaller net tensile areas and lower strength compared to those with single-row holes [10]. Progressive damage analysis was used to study the failure and damage of laminates [11,12,13]. The progressive damage model was applied based on the Hashin criteria to analyze the failure of notched laminates. This model successfully evaluated the damage and predicted the ultimate tensile strength for different lay-ups by combining it with nonlinear FEA for laminate systems with circular holes under tension [14]. The damage and failure of composite laminates under stress have been studied using various models, such as continuum damage mechanics [15,16] and FEA combined with the extended finite element method, which has proven to be a simple and effective technique for practical applications [17,18,19].

In 2021, the nominal strength of an open-hole specimen was predicted using a non-destructive experimental method based on the natural vibration of composite laminates [20]. The core idea of this method was to utilize natural vibrations induced by a lobster blow in an extracted model to calculate the mechanical and fracture properties of composite laminates. Developing a model that can quickly predict the behavior of specimens with holes is crucial for designers to select appropriate materials for specific applications.

Most of the previous studies have focused on predicting tensile strength, whereas a reliable model for predicting failure strength in compression is scarcely available. Therefore, this work was conducted to achieve three main objectives: (1) to predict the size effect in composite laminates with central loading using the progressive damage model, (2) to predict the nominal strength of composite plates with two holes in the longitudinal and transverse directions, considering different distances between the holes, and (3) to investigate the effect of an additional hole on strength reduction or stress relief.

The structure of this paper is as follows: the Section 1 presents the finite element model, the Section 2 outlines the progressive damage model, the Section 3 explains the cohesive laws, and finally, the results and discussion are provided.

## 2. Progressive Damage Model

### 2.1. Hashin Damage Model

There are four different failure modes proposed by Hashin. These modes are as follows: fiber compression, fiber tension, and matrix cracking due to compression or tension [21]. The modes are as follows:

Mode I: Fiber tension

(1) $$F_{1}={\left(\frac{{\bar{\sigma }}_{11}}{\chi^{Τ}}\right)}^{2}+\alpha \;{\left(\frac{{\bar{\sigma }}_{11}}{S^{Τ}}\right)}^{2},\;\mathrm{where}\;0\leq \alpha \geq 1$$

Mode II: Fiber compression

(2) $$F_{1}={\left(\frac{{\bar{\sigma }}_{11}}{\chi^{C}}\right)}^{2}$$

Mode III: Matrix tension

(3) $$F_{111}={\left(\frac{{\bar{\sigma }}_{22}}{Y^{Τ}}\right)}^{2}+\alpha \;{\left(\frac{{\bar{\sigma }}_{12}}{S^{Τ}}\right)}^{2}$$

Mode IV: Matrix compression

(4) $$F_{1V}={\left(\frac{{\bar{\sigma }}_{22}}{2S^{Τ}}\right)}^{2}+\alpha \left[{\left(\frac{{\bar{Y}}_{C}}{{2S}^{Τ}}\right)}^{2}-1\right]\left(\frac{{\bar{\sigma }}_{22}}{2{\bar{Y}}_{C}}\right)+{\left(\frac{{\bar{\sigma }}_{12}}{S^{L}}\right)}^{2}$$

where ${\bar{\sigma }}_{11}$, ${\bar{\sigma }}_{22}$, and ${\bar{\sigma }}_{12}$ are principal stress components at each ply and $Y^{Τ}$, $\chi^{C}$, ${\bar{Y}}_{C}$, $S^{L}$, and $S^{T}$ are composite laminate strengths. The material’s elastic and mechanical properties are listed in Table 1 and Table 2, which were considered the most important properties for implementing Hashin’s damage criteria in the FEM. This model was not a standalone model as it cannot predict the failure of fiber–matrix interaction accurately [22,23], unless it is combined with other damage evaluation laws or degradation rules [24].

### 2.2. Damage Evaluation Criteria

**Figure 1:**
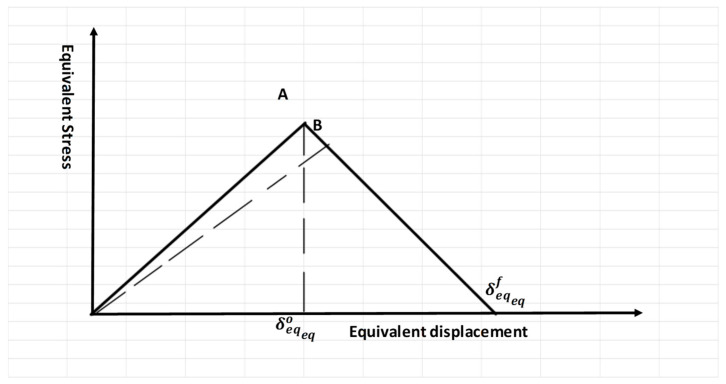
Linear damage law. A: The slope of the first line. B: The second slope.

Every failure involves crack initiation and crack growth, both of which are governed by the principles of damage development. Crack initiation is described by the Hashin model, which assumes a linear response. Once failure starts, the damage initiation criteria are applied according to the damage evolution laws, which also reduce the need for refining mesh sizes [27,28,29]. In Figure 1, a typical linear tensile separation response is shown, as defined by the four failure modes of Hashin’s progressive damage criteria. The slope of the first line represents the start of damage, corresponding to the elastic region. The second slope, which is negative, indicates failure beyond the elastic region, commonly referred to as the softening behavior in polymers.

The variable D can be calculated as follows:

(5) $$D=\frac{\delta_{eq}^{f}\left(\delta_{eq}-\delta_{eq}^{o}\right)}{\delta_{eq}\left(\delta_{eq}^{f}-\delta_{eq}^{o}\right)}$$

where D represents the damage variable factor and $\delta_{eq}^{o}$ and $\delta_{eq}^{f}$ are the equivalent displacements at the end of initiation and the end of failure, respectively.

A sudden degradation rule proposed by Xiao and Ishikawa [24] was implemented immediately after a failure occurred. This rule was designed to prevent the inner ply from carrying specific loads [30].

### 2.3. Cohesive Zone Model

**Figure 2:**
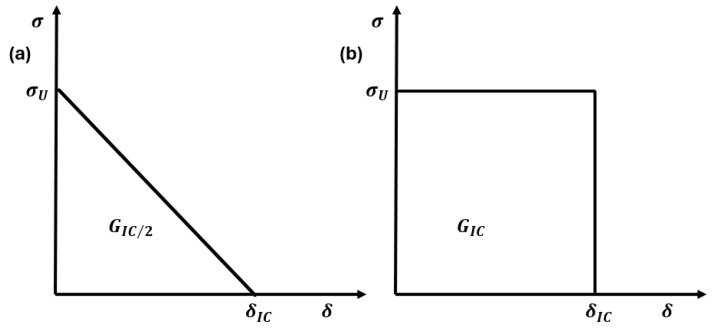
Cohesive zone laws illustrating the relationship between traction and separation: (**a**) linear cohesive law, which shows a linear decrease in traction with increasing separation, and (**b**) constant cohesive law, where the traction remains constant up to a critical separation point.

The cohesive zone model was developed based on cohesive stress at the crack tip and the crack opening displacement (δ). It is commonly referred to as the softening function for quasi-brittle materials, such as tough ceramics, ice, and composite laminates. A specimen fails when the crack opening displacement reaches the critical value ($\delta_{C}$). The most commonly used cohesive laws are two-parameter cohesive laws, which depend on the surface release energy (G_IC_) and the stress at the crack tip ($\sigma_{C}$) [1]. The cohesive zone model distributes the stored energy in the material around the crack tip. Examples of the most common cohesive laws, i.e., the linear and constant cohesive laws, are shown in Figure 2a,b.

### 2.4. Strength Prediction

**Figure 3:**
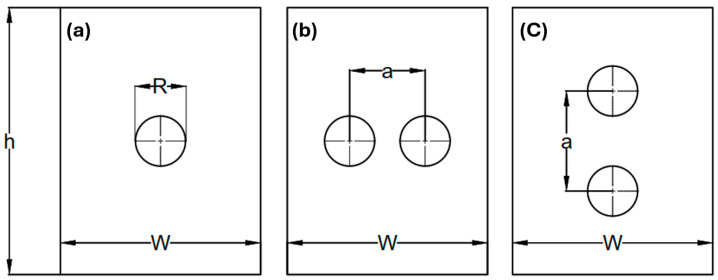
Plate with two holes: (**a**) central hole, (**b**) transversely located hole, and (**c**) longitudinally located hole.

The problem considered was a composite plate with either a central hole (see Figure 3a), two holes arranged in the transverse direction (Figure 3b), or two holes in the longitudinal direction at a distance (a) apart (Figure 3c). To determine the nominal strength of the composite laminates, the cohesive zone law was applied, using a constant and linear model for specimens with only central holes. Subsequently, a correction factor $f\left(a/d\right)$, accounting for the spacing between holes, was calculated. This allowed the nominal strength of composite plates with multiple holes to be calculated. For the complete derivation of the law implementation, please refer to papers [1,7,31].

A brief description is provided below.

The generalized cohesive nominal strength equation can be measured as follows [31]:

(6) $$S=\sum_{i=1}^{n}\beta_{i}\sigma_{i}$$

(7) $$\beta_{i}=-\frac{2}{\pi }\left[\sin^{-1}\left(\frac{c_{i}}{d_{s}}\right)-\sin^{-1}\left(\frac{b_{i}}{d_{s}}\right)\right]f\left(R/w\right)$$

where $f\left(R/W\right)$ is a correction function and $c_{i}$ and $b_{i}$ are displacements at specific points (i) on the crack face found in refs. [1,7,31].

For a constant cohesive law, as shown in Figure 2b, the nominal strength can be calculated using Equation (8). In this case, the stress on the crack face (cohesive stress) is equal to the unnotched tensile strength ($\sigma_{u}$) of the composite laminates.

(8) $$\sigma_{N}=\frac{\beta_{i}\sigma_{u}}{\beta_{P}}$$

where $\beta_{P}=1-2R/W$ is the correction factor at the plastic limit. The cohesive stress distribution the over crack face $(\sigma_{i}$) for the linear cohesive law, as shown in Figure 2a, can be calculated using the equation completely derived in [31] and modified with the size effect law in [7].

(9) $$\sigma_{i}=\sigma_{u}\left\{1-\frac{\sigma_{u}\delta_{i}}{2\;G_{C}}\right\}$$

Returning to Equations (6) and (9), the cohesive stress distribution for the linear cohesive law can be calculated using the following Equation (10) [31]:

(10) $$\sigma_{i}=W_{C}{\left\{\frac{\delta_{C}}{\sigma_{u}}\delta_{ij}+f_{ij}\right\}}^{-1}I_{1}$$

where $\delta_{C}$ represents the critical crack opening and $f_{ij}$ represents the connection function completely calculated in refs. [1,7,31,32].

## 3. Finite Element Model and Experimental Validation

**Figure 4:**
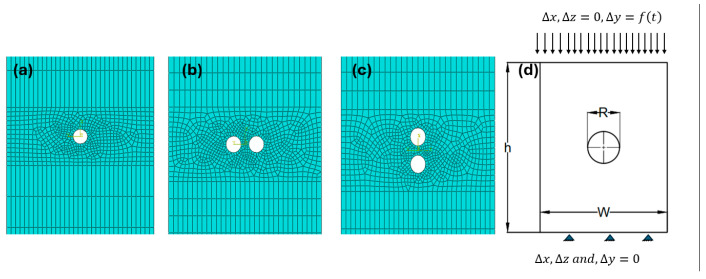
Top view of the 3D model showing: (**a**) the plate with a central open hole, (**b**) two transverse holes, (**c**) two longitudinal holes, and (**d**) the boundary condition domain.

The composite material domain consisted of a rectangular 3D plate with central holes, as shown in Figure 4a, and another configuration with two holes arranged in the transverse and longitudinal directions, illustrated in Figure 4b and Figure 4c, respectively. The distance between the two holes (a) ranged from 3.5 d to 7.5 d, where d, the hole diameter, was 5 mm according to [6]. The sample domain was modeled as a continuum shell, consisting of 3074 elements for the transverse holes and 2974 elements for the longitudinal holes, as shown in Figure 4b and Figure 4c, respectively. A linear SC8R element type was used with dynamic explicit step loading, along with hourglass control and element deletion criteria. A tabulated amplitude was applied. For the sample with a central hole, as shown in Figure 4a, which was used to predict the size effect and validate the model accuracy, the central hole diameters were varied at 5 mm, 7 mm, 10 mm, and 15 mm. The same mesh area was used for all configurations, with a total of 3346, 3330, 3230, and 2962 elements, respectively. The load was applied to one end of the plate, whereas the opposite end was fixed, as illustrated in Figure 4d.

**Figure 5:**
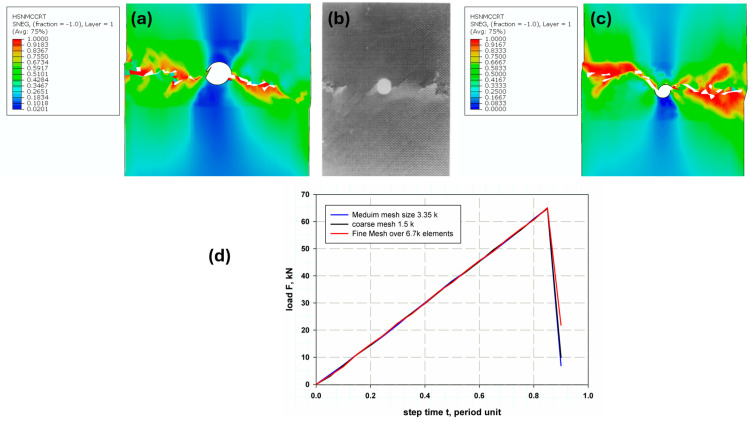
Mesh convergence study with elements size: (**a**) present model with 3k elements, (**b**) experimental [6], (**c**) 6.7k elements, and (**d**) varying mesh size load-carrying capacities.

According to Figure 5, which compares three different mesh densities—fine (6692 elements), medium (3346 elements), and coarse (1512 elements)—and studies by the authors [33,34], it was confirmed that the model is independent of mesh refinement. This is because elastic materials exhibit a continuous and uniform stress–strain behavior, ensuring convergence even with coarser meshes. Additionally, the linear stress–strain relationship in elastic materials reduces sensitivity to mesh density.

The primary objective of this study was to evaluate the bearing capacity of composite laminates rather than the mechanisms of delamination behavior. Although finer meshes can capture more detailed crack paths and ply failures, the chosen mesh density was sufficient to achieve accurate results for global bearing capacity, which was the main focus of this investigation. Figure 5a shows the damage contour for the medium mesh compared with the failure mode observed in an experimental study [6] (Figure 5b). In contrast, Figure 5c presents the results for a finer mesh with more than 6000 elements. The differences between the two have minimal impact on accuracy, whereas the medium mesh significantly reduces computational time and cost. This can be attributed to the implementation of the damage evaluation criteria in the model, which ensures reliable results even with moderate mesh densities (see Figure 5d).

The mechanical properties were extracted from composite laminates of T800/924C carbon/epoxy with a stacking sequence [(±45/0_2_)_3_]_s_ of 24 layers. It was cut into a plate with a thickness of 3.1 mm, a width of 50 mm, a total length of 245 mm, and a gauge length of 145 mm [6]. The holes should be arranged along the axis of symmetry of the plate, either in the longitudinal or transverse direction, ensuring that R ≤a≤w, as described in [6].

## 4. Results and Discussion

### 4.1. Finite Element Results and Cohesive Law Models

**Figure 6:**
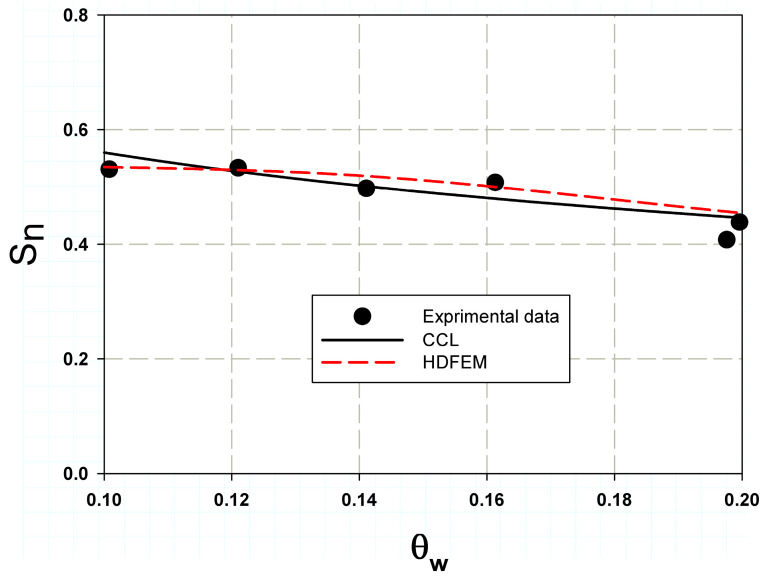
HDFEM validation with the experimental results of Soutis and Fleck [31] for composite laminates with varying hole diameters (varying aspect ratios).

**Figure 7:**
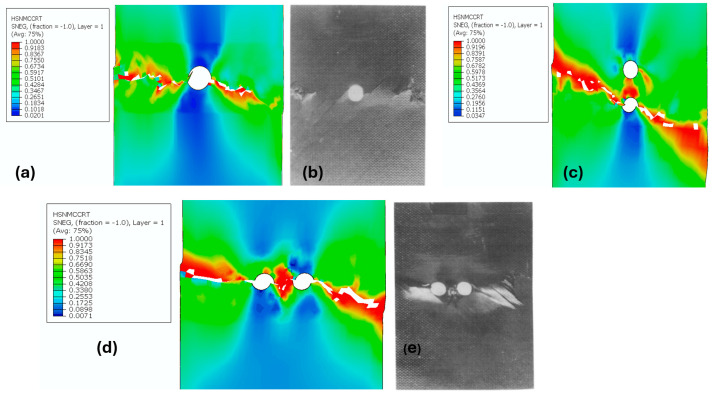
Damage predicted using HDFEM for (**a**) central holes, (**b**) experimental data [6], (**c**) two longitudinally aligned holes, (**d**) two transversely aligned holes, and (**e**) experimental data [6].

Figure 6 shows the predicted nominal strength of the open-hole composite specimens with varying aspect ratios ($\theta_{W}$). It is clearly observed that strength decreases as the aspect ratio increases, which is known as the size effect or scaling effect [31]. The main objective of simulating the central hole was to validate the Hashin Damage Finite Element Model (HDFEM) with experimental data and the two-parameter cohesive laws, including the constant cohesive law described in Equation (8). The matrix cracking contour and fracture surface predicted by Hashin were similar to those observed in the experimental study by Soutis and Fleck [31], as shown in Figure 7. The maximum stress concentration was clearly observed around the holes, with a crack length proportional to R. The prediction accuracy was over 95% on average, as seen in Figure 6.

**Figure 8:**
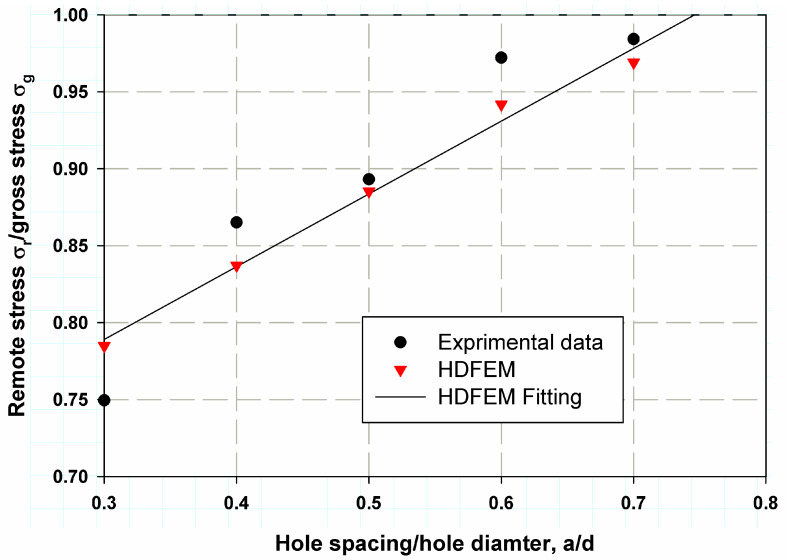
HDM validation with the experimental work of Soutis et al. [6]. for composite laminates with two transverse holes.

**Figure 9:**
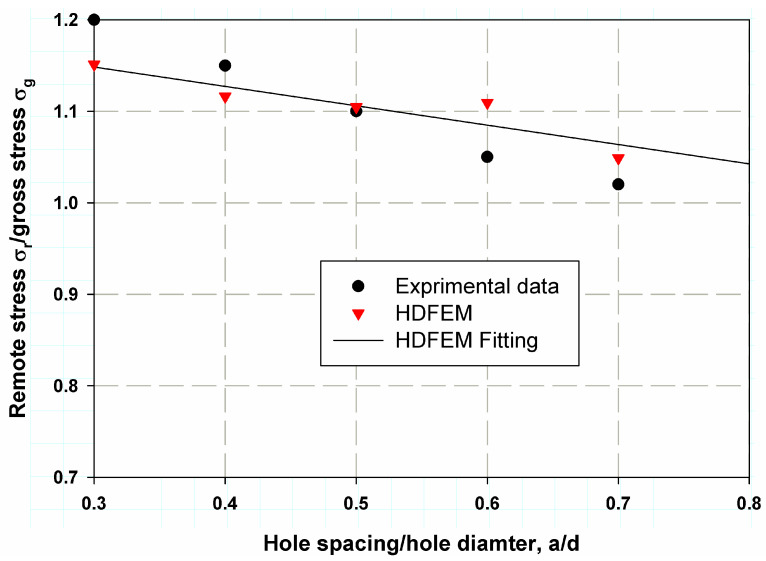
HDM validation with the experimental work of Soutis et al. [6]. for composite laminates with two longitudinal holes.

Table 3 and Figure 8 show the predicted nominal stresses compared to the experimental data for composite laminates with two aligned transverse holes. The model was found to be very close to the experimental data from Soutis et al. [6]. The percentage error was low (4.72% for the sample with 1.5d spacing between holes), and the smallest error was 0.88% for the case with 2.5 d spacing. The average percentage error was 2.7%, indicating good accuracy and precision in model validation. A fitting linear regression was proposed, which provided resonance data that can be used to determine the correction function $f\left(a/d\right)$ in Equations (11)–(14). The same trend was observed for two longitudinally aligned holes, as shown in Table 4 and Figure 9. The maximum percentage error was 5.65% for the case with a larger spacing ratio of 3, whereas the minimum percentage error was 0.42% and the average percentage error was 3.18%. A predictive model is crucial as it helps to make informed decisions, leading to cost savings, higher efficiency, or better results, depending on the application. The percentage error for transversely aligned holes was lower than for longitudinally aligned holes due to more uniform stress distribution, simpler damage mechanisms, and a better match between the assumptions of the damage model and the actual material behavior. In contrast, the more anisotropic stress concentrations and complex damage mechanisms associated with longitudinally aligned holes make the model’s predictions less accurate, resulting in a higher percentage error [35,36].

**Figure 10:**
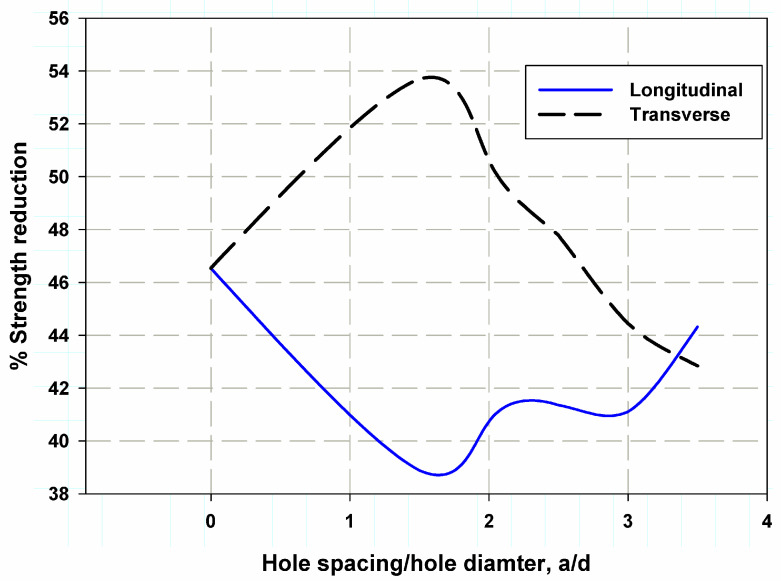
Stress reduction influenced by the insertion of an extra hole.

The failure modes were simulated through the holes, with the Mises stress shown as a red-colored stress concentration region (see Figure 7). Figure 10 illustrates the effects of existing holes on stress distribution and nominal strength. It was clear that adding additional holes in the longitudinal direction of the central holes reduced the overall strength of the plate. The percentage of strength reduction varied with the distance between the holes. However, for transverse holes, the trend was the opposite, as increasing the distance between the holes resulted in an increase in strength.

This is because, with additional holes in the longitudinal direction near the central holes, the stress concentration factor becomes less complex, and the structure is strengthened by an increase in load-bearing capacity, particularly with closer spacing. On the other hand, with transverse holes, a greater distance between the holes reduces stress interaction and concentration, allowing for a more uniform stress distribution, which can potentially increase the strength of the plate.

### 4.2. Nominal Strength of the Plate with Multiple Holes

The nominal strength of the composite plate with two open holes subjected to compression load can be predicted using the correction function $f\left(a/d\right)$, which was obtained by HDFEM fitting through linear regression, as previously discussed.

Constant cohesive law (CCL):

For the constant cohesive law, Equation (8) can be multiplied by the correction factor obtained from curve fitting of the data in Figure 8 and Figure 9, as follows:

Transverse direction:

(11) $$\sigma_{N}=\frac{\beta_{i}\sigma_{u}}{\beta_{P}}f\left(\frac{a}{d}\;\right)\;$$

where the correction function is given by:

(12) $$f\left(\;\frac{a}{d}\right)=\left(0.47\;\left(\frac{a}{d}\right)+0.64\right)$$

Longitudinal direction:

(13) $$\sigma_{N}=\frac{\beta_{i}\sigma_{u}}{\beta_{P}}\bar{f}\left(\frac{a}{d}\;\right)\;$$

where the correction function is calculated as:

(14) $$\bar{f}\left(\frac{a}{d}\;\right)=\left(-0.21\left(\frac{a}{d}\;\right)+1.21\right)$$

Linear cohesive law (LCL):

For the linear cohesive law, Equation (6) can be multiplied by the correction factor obtained from curve fitting of the data in Figure 8 and Figure 9, as follows:

Transverse direction:

(15) $$S=\sum_{i=1}^{n}\beta_{i}\sigma_{i}f\left(\frac{a}{d}\;\right)$$

Longitudinal direction:

(16) $$S=\sum_{i=1}^{n}\beta_{i}\sigma_{i}f\left(\frac{a}{d}\;\right)$$

**Figure 11:**
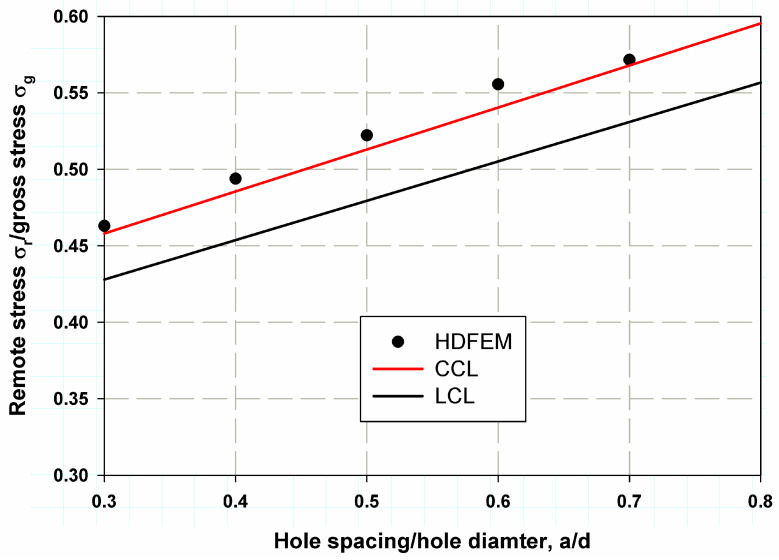
Model validation comparing the predicted and HDFEM results for a structure with two transverse holes.

**Figure 12:**
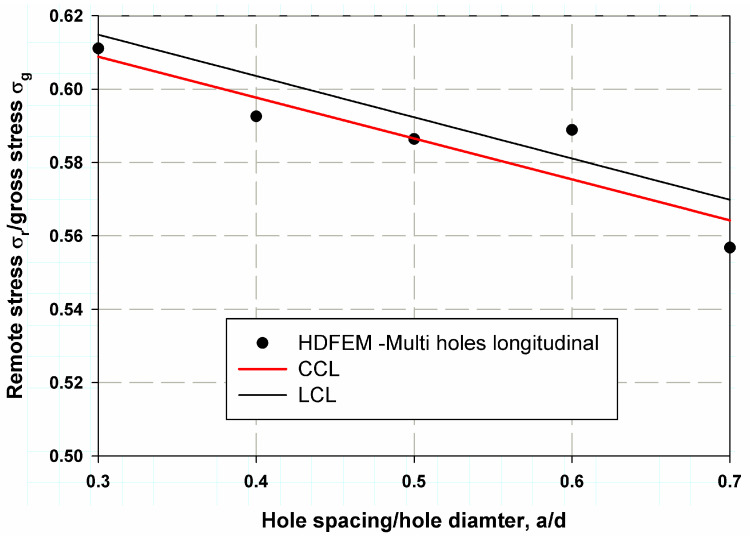
Model validation comparing the predicted and HDFEM results for a structure with two longitudinal holes.

Figure 11 and Figure 12 show the predicted nominal strength for two transversely oriented holes using the proposed models based on the following cohesive laws: LCL and CCL. CCL was found to provide more accurate data and predictions than LCL for both transversely and longitudinally oriented holes. The average error for CCL was 2.5%, whereas for LCL, it was 9% when the two holes were transversely aligned, as shown in Figure 10.

However, in the case of two longitudinally aligned holes, as observed in Figure 11, the percentage error for LCL was larger than that for CCL. Specifically, the error was 1.2% for LCL and 0.5% for CCL. The difference between LCL and CCL can be attributed to several factors. The first reason is the surface release energy, where the linear cohesive law assumes a varying pressure–separation relationship, reflecting the increasing resistance of the material as the crack tip approaches the failure point. In contrast, the constant cohesive law assumes a constant compressive force during crack propagation, resulting in a simpler and typically higher nominal strength prediction. This difference in crack growth behavior explains the observed differences in the predicted strengths [37]. Additionally, the symmetrical holes, which were oriented transverse to the grain direction, led to a more uniform stress distribution in the specimens.

## 5. Conclusions

The Hashin Damage Finite Element Model (HDFEM), also known as the progressive damage model, predicts the size effect on the compressive response of a composite plate with scaled geometry. Additionally, the progressive damage model helps to determine the correction factor for predicting the nominal strength of composite plates with multiple aligned holes in both transverse and longitudinal directions. The model provides an average prediction accuracy of 2.5%.

The proposed model, based on the linear and constant cohesive laws with two parameters, provides highly accurate results, with a minimum average error of 0.5% in the case of the linear cohesive law for two holes aligned in the longitudinal direction. The presence of additional holes in the longitudinal direction decreases the overall strength ratio. It acts as a stress relief factor, reducing stress concentration around the central hole region. Specifically, the percentage reduction in strength increases to 17% in the case of two holes arranged in the longitudinal direction.

The model provides valuable tables and diagrams that are important for design considerations and material selection.

## Figures and Tables

**Table 1:** Elastic constant of T800/924C carbon fiber/epoxy [25].

Properties	E_1_ (GPa)	E_2_ (GPa)	E_3_ (GPa)	µ_13_	µ_12_	µ_23_	G_12_ (GPa)	G_13_ (GPa)	G_23_ (GPa)
Value	161	9.25	9.25	0.34	0.34	0.34	6	6	56

**Table 2:** Mechanical properties and fracture toughness of [(±45/0_2_)_3_]_2s_ carbon fiber composite laminates [6].

Properties	${Χ}^{T}$ MPa	${Χ}^{C}$ MPa	Yc MPa	$S^{L}$ MPa	$S^{T}$ MPa	(G_IC_)t kJ/m^2^	(G_IC_)c kJ/m^2^	(G_IC_)tM kJ/m^2^	(G_IC_)cM kJ/m^2^
Value	1615	63	225	109 [26]	109	31	31	0.575	50.575

**Table 3:** HDFEM validation for the specimen with two transverse holes, compared with the experimental work of Soutis et al. [6].

a/d	$\mathrm{Nominal}\;\mathrm{Stress}\;\sigma_{r}$, MPa [6]	HDFEM	% Error
1.5	358	375	4.72
2	413	400	−3.23
2.5	427	423	−0.88
3	465	450	−3.12
3.5	470	463	−1.54

**Table 4:** HDFEM validation for the specimen with two longitudinal holes, compared with the experimental work of Soutis et al. [6].

a/d	$\mathrm{Nominal}\;\mathrm{Stress}\;\sigma_{r}$, MPa [6]	HDFEM	% Error
1.5	516	495	−4.07
2	494.5	480	−2.93
2.5	473	475	0.42
3	451.5	477	5.65
3.5	438.6	451	2.83

## Data Availability

Data are contained within the article.

## List of Nomenclature

Symbols	Definitions
$\theta_{W}$	Aspect ratio equals R/w
$\sigma_{C}$	Cohesive stress
$\sigma_{i}$	Component of stress for the *i*-th element on the crack face
$Y^{Τ}$ $,\;\chi^{C}$ $,\;{\bar{Y}}_{C}$ $,\;S^{L}$ $\mathrm{and}\;S^{T}$	Composite laminate strengths
$f_{ij}$	Connection function calculated for the element i, j
$\beta_{P}$	Correction factor at the plastic limit
$f\left(a/d\right)$	Correction factor for spacing between holes
$\delta_{C}$	Critical opening displacement
D	Damage variable factor
$c_{i}$ $,\;b_{i}$	Displacements at specific points (i) on the crack face, which are equal to $c_{i}=\left(R+\iota \right)-\left(\frac{i-1}{n}\right)$ $b_{i}=\left(R+\iota \right)-\left(\frac{i}{n}\iota \right)$
E_1_, E_2_, E_3_, µ_12,_ µ_13,_ µ_23_	Elastic constants for T800/924C carbon fiber/epoxy
$\delta_{eq}^{o}$ $,\;\mathrm{and}\;\delta_{eq}^{f}$	Equivalent displacement at the initiation and failure stages
$F_{1}$ $.\;F_{11}$ $,\;F_{111}$ $,\;\mathrm{and}\;F_{1V}$	Functions represent fiber tension, fiber compression, matrix tension, and matrix compression
R	Hole radius
$\delta_{ij}$	Kronecker delta in continuum mechanics
$d_{s}$	Length from hole centers to the crack tip, which is equal to $d_{s}=l+R$
l	Length of micro-buckled region
$S_{n}$	Nominal strength
δ	Opening displacement
w	Plate width
${\bar{\sigma }}_{11}$ $,\;{\bar{\sigma }}_{22}$ $\mathrm{and}\;{\bar{\sigma }}_{12}$	Principal stress components at each ply
$\sigma_{r}$	Remote strength or nominal strength (Sn)
$\beta_{i}$	Shape factor
G_IC_	Surface release energy
$\sigma_{u}$	Un-notch composite strength
$f\left(R/w\right)$	Width and hole correction function
